# Enteropathy-associated t-cell lymphoma presenting as refractory cutaneous ulcers in a 28-year-old male: a case report and literature review

**DOI:** 10.3389/fonc.2025.1716730

**Published:** 2025-11-21

**Authors:** Dejie Zhao, Quanlin Li, Dan Liu, Jie Xu, Zheng Liu, Yanan Zhao, Zhixin Cheng, Ming Liu

**Affiliations:** 1 The First Clinical Medical College, Shandong University of Traditional Chinese Medicine, Jinan, China; 2 Department of Medical Imaging, Affiliated Hospital of Shandong University of Traditional Chinese Medicine, Jinan, China; 3 Department of Pathology, Beijing Chaoyang Integrative Medicine Rescue and First Aid Hospital, Beijing, China; 4 Department of Hematology, Affiliated Hospital of Shandong University of Traditional Chinese Medicine, Jinan, China; 5 Department of Vascular Surgery, Affiliated Hospital of Shandong University of Traditional Chinese Medicine, Jinan, China

**Keywords:** enteropathy-associated T-cell lymphoma, cutaneous ulcer, celiac disease, paraneoplastic syndrome, misdiagnosis, young patients, case report

## Abstract

**Background:**

Enteropathy associated T-cell lymphoma (EATL) is a rare and aggressive subtype of peripheral T-cell lymphoma, most commonly associated with celiac disease (CD). It typically presents with gastrointestinal symptoms and carries a poor prognosis. Cutaneous involvement as the initial manifestation, particularly in the form of refractory skin ulcers in young patients, is rare.

**Case summary:**

We report a 28-year-old male who presented with a one-year history of refractory, non-healing ulcers on the right foot and several years of chronic diarrhea, initially misdiagnosed and treated as a chronic infection in April 2019. Despite multiple antibiotic regimens, surgical debridement, and immunosuppressive therapy, the lesions progressed symmetrically to involve the right hand and left thigh. The patient underwent amputation of the right lower limb and right hand in October 2020. One month later, the patient developed acute peritonitis secondary to jejunal perforation. Laparotomy revealed a transmural tumor mass, and histopathological and immunohistochemical analyses confirmed EATL (CD3+/CD103+/TIA-1+/GRANB+, CD5-/CD8-, Ki-67 ~70%). The patient died of multiorgan failure three weeks postoperatively in December 2020.

**Conclusion:**

This case underscores that EATL may manifest with widespread cutaneous ulcers more than one year before the onset of severe gastrointestinal symptoms. In patients with chronic diarrhea and refractory skin ulcers, celiac disease should be excluded, while cutaneous lesions should be recognized as potential paraneoplastic manifestations of an underlying lymphoma. Diagnostic delays—driven by anchoring bias and insufficient tissue sampling—are critical contributors to advanced disease at presentation and poor clinical outcomes.

## Introduction

EATL is a rare and highly aggressive peripheral T-cell lymphoma arising in the context of long-standing CD (previously termed refractory CD type I) (1, 2). With an estimated annual incidence of less than 1%, and a five-year survival rate ranging from 11% to 20%, EATL predominantly affects older adults (ages 60–70) in Western populations and is strongly associated with a history of CD (3–5). The classic clinical presentation includes severe abdominal pain, weight loss, malabsorption, and complications such as bowel obstruction or perforation (2). While hematogenous dissemination occurs late in disease progression, cutaneous involvement remains infrequent and usually signifies advanced-stage metastatic disease (6). The presentation of EATL with progressive, treatment-refractory cutaneous ulcers as the sole initial manifestation, especially in a young adult, is rare. Such cases may be easily misdiagnosed as chronic infections, vasculitis, or other inflammatory dermatoses, leading to critical delays in diagnostic. We present a fatal case of EATL in a 28-year-old man whose disease began with recalcitrant skin ulcers, culminating in a 19-month delay in diagnosis. Based on the misdiagnosed case, this article systematically analyzes the causes of diagnostic errors by focusing on disease subclassification, diagnostic criteria, and therapeutic characteristics, and further explores the potential association between idiopathic refractory cutaneous ulcers and hematologic malignancies.

## Case presentation

**Figure 1:**
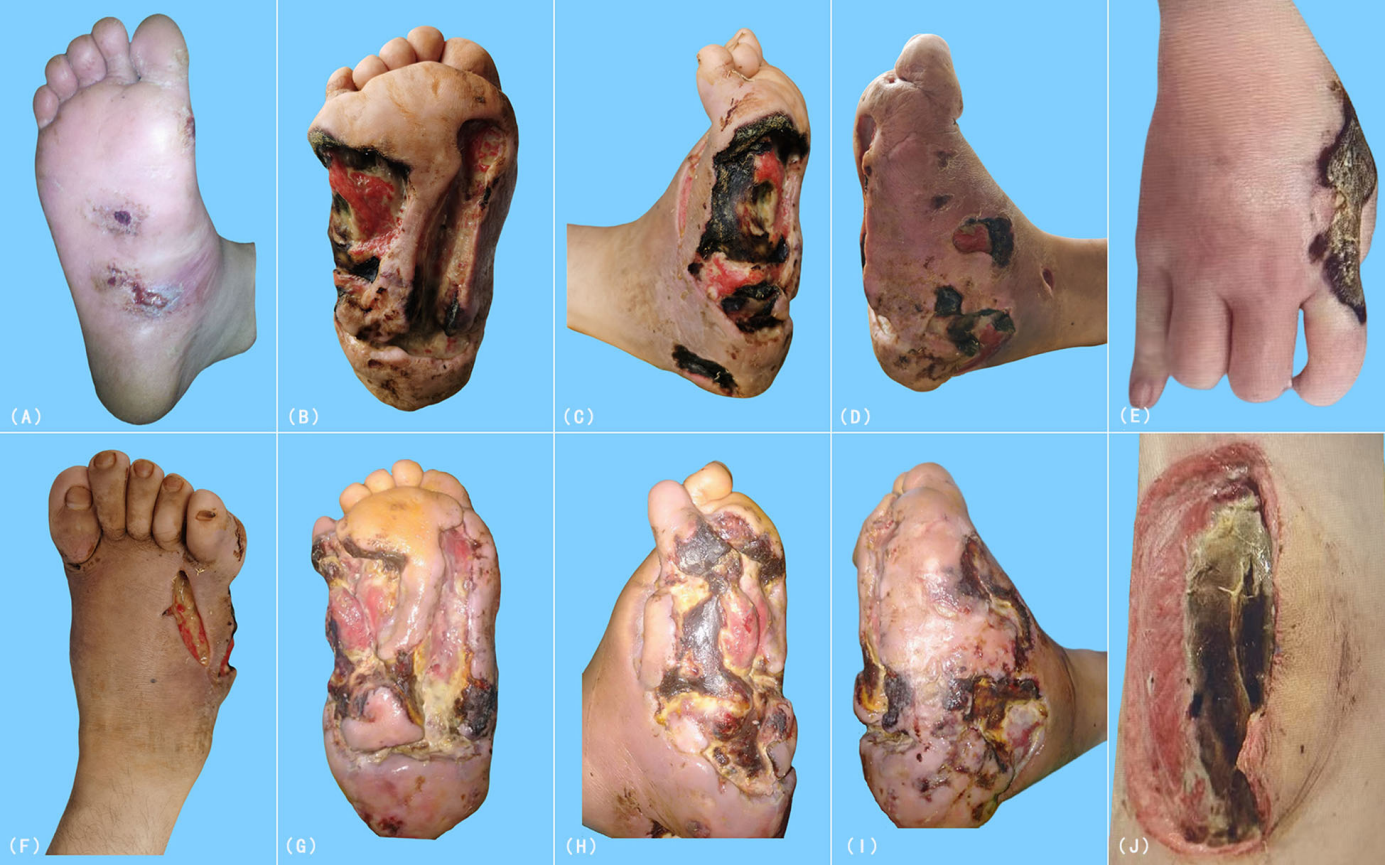
Clinical progression of cutaneous ulcers and necrotic lesions in the patient. **(A)** Persistent ulcer on the plantar surface of the right foot at initial presentation (April 2019). **(B–D)** Progressive enlargement and deepening of the ulcer over one year. **(E)** Necrotic lesion on the right hand in August 2020. **(F–I)** Worsening cutaneous lesions despite treatment. **(J)** Necrotic plaque on the medial aspect of the left mid-thigh.

**Figure 2:**
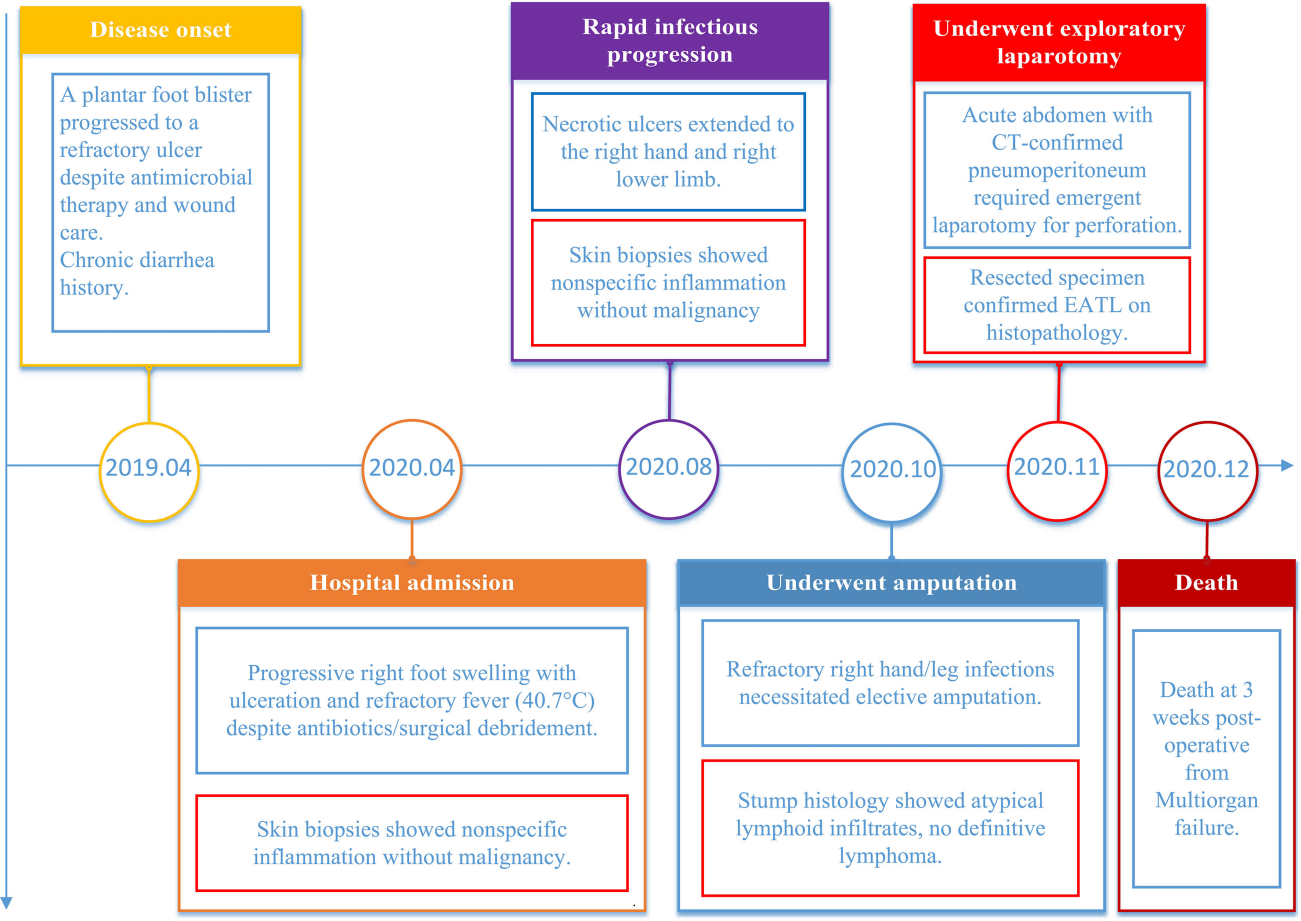
The timeline chart illustrates the progression of the patient’s typical symptoms, key laboratory findings, surgical interventions, and other significant events from onset to mortality.

A previously healthy 28-year-old male presented in April 2019 with a non-healing ulcer on the plantar aspect of the right foot (**Figure 1A**), accompanied by chronic diarrhea of approximately 8 years’ duration. The diarrhea was characterized by exacerbations following the heavy ingestion of wheat-based foods, cold or raw diets, or emotional stressors. The patient’s medical history was also significant for irregular dietary habits. (**Figure 2**). Initially diagnosed as chronic bacterial infection and chronic enteritis, the patient received multiple courses of broad-spectrum antibiotics over a 12-month period, targeting Gram-positive, Gram-negative, and anaerobic bacteria, along with antidiarrheal management and local wound care. Although mild improvement in diarrhea was noted, the cutaneous ulcers showed no clinical response and progressively worsened in both size and depth over the following year (**Figures 1B–D**). A short course of systemic corticosteroids (prednisone equivalent, approximately 0.5–1 mg/kg/day for two weeks) was administered in an attempt to control presumed inflammatory or autoimmune activity, but this intervention provided no benefit and was discontinued due to lack of efficacy. By April 2020, the patient developed systemic symptoms, including high-grade fever (peak temperature 40.7°C), significant weight loss, severe hypoalbuminemia (16.4 g/L), anemia (hemoglobin 81 g/L), and markedly elevated inflammatory markers (ESR 108 mm/h, CRP 36.5 mg/L), indicating progressive systemic disease despite extensive empirical treatment.

**Figure 3:**
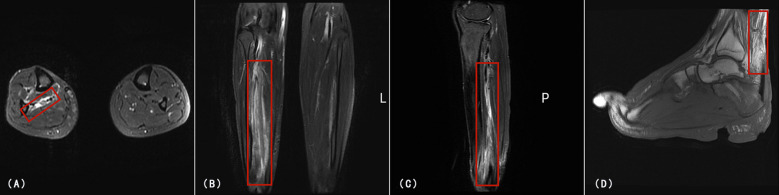
MRI of the right lower limb and foot. **(A)** Axial T2-weighted fat-suppressed image showing high signal intensity(Red rectangle) in the subcutaneous and deep fascial planes of the right lower limb. **(B, C)** Coronal and sagittal T2-weighted images of the lower limb revealing multifocal fluid-like signal(Red rectangle) abnormalities and marked soft tissue edema along vascular and neurogenic pathways. **(D)** Sagittal T2-weighted image of the right foot showing high signal intensity (Red rectangle) in the posterior aspect of the ankle joint, severe soft tissue edema, joint deformity at the interphalangeal articulations, and multiple areas of tissue defect.

Abdominal ultrasonography and CT scans of the chest and abdomen revealed an enlarged spleen without space-occupying lesions. All tumor-associated serum markers were within normal limits. Magnetic Resonance Imaging (MRI) of the lower limb (**Figures 3A–C**) and foot (**Figure 3D**) revealed extensive soft-tissue inflammation extending along vascular planes, while ultrasound showed bilateral inguinal lymphadenopathy with lymph nodes appearing non-tender, regular in shape, and well-demarcated cortical and medullary differentiation, without abnormal hypervascularity on Doppler imaging. These findings raised suspicion for necrotizing fasciitis or systemic vasculitis.

Despite aggressive antimicrobial and immunomodulatory therapies, including corticosteroids, the condition worsened (**Figures 1F–I**). In August 2020, symmetric necrotic lesions developed on the right hand (**Figure 1E**) and left thigh (**Figure 1J**), suggesting a systemic process. Repeat superficial skin biopsies from ulcer margins showed necrotic tissue and dense inflammatory infiltrates, with one report noting “atypical lymphocytes” but no definitive evidence of malignancy.(**Table 1**) The clinical picture was consistent with a treatment-refractory infection or anti-neutrophil cytoplasmic antibody-negative vasculitis (7).

**Table 1:** Details of serial skin biopsies and pathological findings.

Biopsy time	Biopsy site & tissue type	Pathological findings	Key note
2020-04	Right plantar region; superficial necrotic black tissue and secretions	Necrosis and coagulative necrosis	First biopsy; mainly necrotic tissue
2020-05	Right plantar region; superficial granulation tissue of ulcer	Granulation tissue hyperplasia with acute and chronic inflammatory cell infiltration	Follow-up biopsy after initial necrotic tissue clearance
2020-06	Right medial plantar region; black necrotic tissue with a small amount of deep granulation tissue	Necrosis and coagulative necrotic tissue; local abundant lymphocyte aggregation with lymphoid follicle formation	First appearance of significant lymphocyte aggregation
2020-10	Left thigh rectus femoris muscle	Extensive necrosis, granulation tissue hyperplasia, massive inflammation, vascular hyperplasia, transmural vasculitis, segmental fibrinoid necrosis, lymphoid hyperplasia, and partial lymphocyte atypia.	First detection of lymphocyte atypia; key clue for subsequent diagnosis

All biopsies were performed on active lesion margins. Pathological results were confirmed by two senior pathologists to ensure diagnostic consistency.

**Figure 4:**
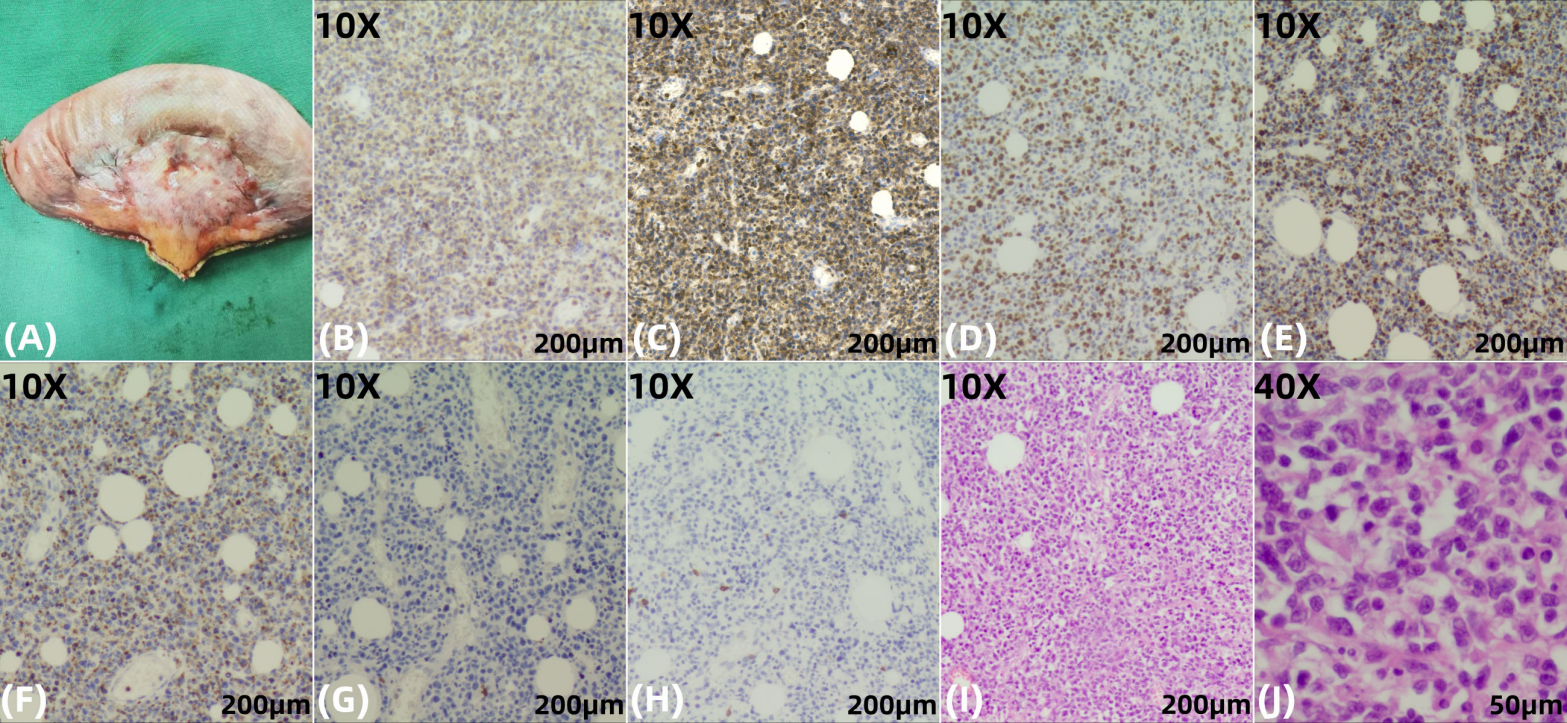
Histopathological and immunochemical findings from the jejunal tumor specimen. **(A)** Gross examination of the resected jejunal segment (length: 15.7 cm) reveals a smooth and intact serosal surface with a gray-white to gray-red discoloration. The mucosal folds are preserved and appear gray-white, with evidence of edema. Adjacent to the intestinal wall, surrounding adipose tissue is replaced by a firm, gray-white to gray-brown, fibrotic-appearing mass measuring approximately 3.5 × 3.5 × 1 cm. **(B, C)**, 10X) The neoplastic cells show diffuse positivity for CD3, confirming their T-cell lineage, and strong co-expression of CD103, a characteristic marker of intestinal intraepithelial lymphocytes, which highly supports the diagnosis of EATL. **(D)**, 10X) High Ki-67 proliferation index (~70%), indicative of an aggressive neoplasm. **(E, F)**, 10X) The neoplastic cells are positive for the cytotoxic granule-associated proteins TIA-1 and Granzyme B, confirming a cytotoxic T-cell phenotype. **(G, H)**, 10X) The tumor cells show loss of CD5 (a pan-T-cell marker) and CD8, a phenotype consistent with EATL. CD5 negativity is particularly significant as it reflects clonal T-cell proliferation, while CD8 negativity helps distinguish Type I EATL from monomorphic epitheliotropic intestinal T-cell lymphoma(MEITL, Type II). **(I, J)**, 10X and 40X) Higher magnification of H&E-stained sections highlighting pronounced cytological atypia and dense neoplastic infiltration within the intestinal wall.

Due to progressive limb necrosis, the patient underwent amputation of the right lower limb and right hand in October 2020. All procedures were performed in compliance with the ethical guidelines established by the Ethics Committee of the Affiliated Hospital of Shandong University of Traditional Chinese Medicine (2025/QT/016) and the Declaration of Helsinki principles. Informed consent was obtained after the risks and procedure were thoroughly explained to the patient and his family. One month later, he developed acute peritonitis. Abdominal CT revealed pneumoperitoneum, indicating bowel perforation. Emergency laparotomy identified a large perforated mass in the jejunum. Histopathology demonstrated transmural infiltration by medium-sized atypical lymphocytes with high mitotic activity and geographic necrosis. (**Table 1**) Immunohistochemistry revealed positivity for CD3, CD43, CD103, TIA-1, and GRANB, negativity for CD5, CD7, CD8, CD20, CD56, and EBER, with a Ki-67 proliferation index of approximately 70% (**Figure 4**). These features were consistent with EATL, Type I (1, 8).

Retrospective review of prior skin biopsies suggested possible early dermal lymphocytic infiltration; however, the superficial depth and restricted horizontal extent of the sampling precluded a definitive diagnosis at that time. Despite surgical resection, the patient developed multiorgan failure and died in December, 2020, three weeks after laparotomy.

## Discussion

Peripheral T-cell lymphomas (PTCLs) constitute a heterogeneous group of aggressive non-Hodgkin lymphomas originating from mature T cells or natural killer (NK) cells. They are broadly classified according to their predominant anatomical involvement into nodal, extranodal, and primary cutaneous subtypes. Among extranodal PTCLs, EATL represents a rare yet clinically significant entity, predominantly originating within the gastrointestinal tract and frequently associated with underlying celiac disease (9). Only 62 cases have been documented globally across 22 medical centers over a 13-year period, constituting 10% to 16% of all primary gastrointestinal lymphomas (4).

EATL is further subclassified into type I (classical, associated with celiac disease and primarily affecting individuals of European ancestry) and type II (monomorphic, less frequently associated with gluten sensitivity and observed predominantly in Asian and Hispanic populations), reflecting distinct clinicopathological and molecular features (1). The most frequent presenting symptoms in EATL patients include diarrhea, followed closely by abdominal pain and gastrointestinal bleeding. Approximately half of patients present with advanced-stage disease at diagnosis (10). Both subtypes are associated with a poor prognosis, exhibiting 5-year survival rates under 20% (11, 12).

The diagnosis of EATL necessitates a comprehensive multimodal approach, integrating histopathological analysis, immunophenotyping, molecular clonality studies, and radiologic and endoscopic findings. However, studies have demonstrated that the diagnostic accuracy of conventional endoscopy is suboptimal, reported as low as 27.3%, with EATL frequently misdiagnosed as Crohn’s disease (10). Histopathological evaluation serves as a cornerstone in the diagnosis. Neoplastic cells typically exhibit CD3, CD30, and CD7 expression, lack expression of CD5, CD8, and CD56, and demonstrate diffuse positivity for cytotoxic granule proteins (TIA-1, granzyme B, perforin) and the epithelial homing integrin CD103 (1). This case demonstrates a classic immunophenotypic profile highly characteristic of EATL type I. The neoplastic cells exhibited diffuse positivity for CD3, confirming their mature T-cell lineage. Notably, they displayed a CD5^-^/CD8^-^ phenotype; the loss of CD5 represents a critical aberrant marker strongly indicative of a neoplastic process, while the absence of CD8 serves to discriminate it from monomorphic epitheliotropic intestinal T-cell lymphoma (MEITL, or EATL type II), which is typically CD8 +. Further supporting the diagnosis was the strong, diffuse positivity for CD103, a hallmark integrin of gut intraepithelial lymphocytes, confirming the mucosal T-cell origin. The co-expression of cytotoxic molecules (TIA-1, Granzyme B) definitively classifies this lymphoma as deriving from a cytotoxic T-cell subset (8). Finally, the high proliferative index (Ki-67 index of 70%) underscores the aggressive nature of this neoplasm, correlating with its characteristically rapid clinical progression. In summary, this immunoprofile (CD3+, CD5-, CD8-, CD103+, cytotoxic proteins+) is diagnostically pivotal for EATL type I, aligning seamlessly with established diagnostic criteria, particularly in the context of a long-standing, overlooked history of chronic diarrhea that may have represented undiagnosed celiac disease—an important predisposing condition for this aggressive lymphoma.

Additionally, Positron Emission Tomography-Computed Tomography (PET-CT) plays a critical role in staging, response assessment, and risk stratification during lymphoma management (13). Advances in molecular pathology and next-generation sequencing technologies have significantly enhanced the efficiency and diagnostic accuracy of EATL through their integration into diagnostic workflows.

Reports of EATL with concurrent cutaneous manifestations are rare, predominantly occurring in elderly patients and manifested as localized ulcerations on the lower extremities and cutaneous hyperpigmentation (14, 15). Another report described lymphoma presenting with dry, black necrosis of the distal fingers and toes in a 68-year-old male, although the subtype was not EATL (16). Additionally, a case of EATL in a 30-year-old female was described, but without any cutaneous involvement (17). To our knowledge, this is one of the youngest reported cases of EATL presenting primarily with cutaneous ulcers. Our case expands the phenotypic spectrum and emphasizes that age alone should not exclude malignancy in the setting of progressive, therapy-resistant skin lesions.

This case underscores a rare and clinically challenging presentation of EATL: refractory cutaneous ulcers as the initial and dominant manifestation in a young adult, preceding gastrointestinal symptoms by 19 months. The diagnosis was only established after a life-threatening complication: bowel perforation, highlighting the risk of delayed recognition when malignancy mimics benign inflammatory or infectious processes. Several factors contributed to the diagnostic delay:

### Atypical clinical phenotype

EATL is extremely rare in patients under 30 years of age (4, 5). It commonly manifests with gastrointestinal symptoms. Therefore, the presentation of cutaneous ulceration as the primary manifestation is highly atypical and susceptible to misdiagnosis. Cutaneous involvement in EATL is typically metastatic and late-stage (6); In this patient, repeated pathological evaluations suggested chronic infection rather than malignancy. Some studies have proposed that this pattern, when appearing as an initial clinical manifestation, could reflect a paraneoplastic process rather than direct tumor spread (18).The same principle applies to a 70-year-old woman who initially presented with cutaneous erythema, in whom rare paraneoplastic keratoderma (Bazex syndrome) was diagnosed after an 8-month delay (19). Notably, in this patient, repeated pathological evaluations at multiple time points consistently indicated chronic infection rather than malignancy, providing no current evidence to support a diagnosis of paraneoplastic syndrome. This misinterpretation may have led clinicians to focus predominantly on the refractory skin ulcers as the dominant clinical feature, thereby overlooking another atypical yet critical manifestation—chronic diarrhea. Although a paraneoplastic etiology cannot be entirely excluded, a more plausible explanation is that the patient’s chronic diarrhea stemmed from unrecognized celiac disease, a well-established precursor to EATL.

### Diagnostic anchoring bias

The initial impression of infection persisted despite therapeutic failure, preventing timely escalation of the differential diagnosis. Each negative superficial biopsy reinforced this misdiagnosis, reflecting cognitive bias common in complex cases (20).

### Inadequate tissue sampling

Repeated superficial biopsies failed to capture deeper dermal or subcutaneous lymphomatous infiltration. A similar case has been reported in which initial misdiagnosis occurred due to shallow biopsy depth (21). In lymphoproliferative disorders, malignant cells may be sparse and masked by a reactive inflammatory background. Deep, full-thickness, and repeat biopsies from active lesion borders are essential for accurate diagnosis (22–24). During exploratory laparotomy, routine biopsy sampling from the terminal ileum and the horizontal (third) portion of the duodenum would help comprehensively assess the extent of intestinal involvement and identify potential celiac disease–associated enteropathic changes, even in the absence of overt mucosal abnormalities. However, due to the emergent setting, biopsies were not taken.

### Unrecognized celiac disease and fragmented care

The patient had a history of chronic diarrhea, suggestive of underlying CD—a well-established predisposing condition for EATL. However, the transient improvement of symptoms following antibiotic therapy led to a misdiagnosis of bacterial enteritis. Moreover, abdominal ultrasound and CT did not reveal any space-occupying lesions, which collectively precluded further comprehensive gastrointestinal evaluation, including contrast-enhanced abdominal CT, gastroscopy, and enteroscopy. Retrospective analysis indicates that the persistent gastrointestinal manifestations likely reflected not only undiagnosed celiac disease but also possible early neoplastic infiltration of the intestinal mucosa. This evolution highlights the significant diagnostic challenge in distinguishing between inflammatory, autoimmune, and premalignant disorders in clinically complex presentations.

Crucially, specific diagnostic tests for CD—such as serum anti-tissue transglutaminase immunoglobulin A (anti-tTG IgA), endomysial antibodies (EMA), and duodenal biopsy—were not performed, contributing to a substantial diagnostic delay (25, 26). Notably, in 20% to 73% of EATL cases (2, 12), the lymphoma precedes the diagnosis of CD, underscoring the high prevalence of unrecognized celiac disease in clinical practice. Furthermore, the lack of a multidisciplinary approach involving gastroenterology and dermatology hindered the integration of systemic clues—particularly the co-occurrence of refractory cutaneous ulcers and chronic gastrointestinal symptoms—that might otherwise have raised earlier suspicion of a paraneoplastic or lymphoproliferative disorder.

Currently, no standardized treatment guidelines exist for EATL. Multimodal therapeutic strategies, incorporating surgery, radiotherapy, chemotherapy, and targeted therapies, constitute the cornerstone of management. For patients with locally advanced small intestinal lymphoma comparable to the present case, surgical resection is indicated when the histological diagnosis remains indeterminate or when complications such as intestinal obstruction, hemorrhage, or perforation occur (27). In this particular patient, surgery was carried out in the setting of intestinal perforation, at which point the disease had advanced to an end - stage state, thus precluding the possibility of timely initiation of chemotherapy. A meta - analysis has revealed that early surgical intervention followed by adjuvant chemotherapy significantly extends 3 - year and 5 - year overall survival, as well as 3 - year progression - free survival, in comparison to chemotherapy alone (28), once again highlighting the crucial significance of early diagnosis.

The CHOP regimen (cyclophosphamide, doxorubicin, vincristine, and prednisone) is regarded as the standard first - line chemotherapy for EATL; however, it is associated with a median overall survival of merely 7 months (12, 29). Rituximab combined with CHOP has also exhibited favorable outcomes in improving progression - free survival, event - free survival, disease - free survival, and overall survival in certain subtypes of T - cell lymphoma (30). The EPOCH regimen (etoposide, prednisone, vincristine, cyclophosphamide, and doxorubicin) shares a similar core but may provide enhanced efficacy in follicular lymphomas (31).For the majority of aggressive intestinal lymphomas, the current standard of care generally entails systemic chemotherapy combined with radiotherapy. A large prospective study has indicated that a multimodal treatment approach, stratified according to disease stage, histological subtype, and surgical resectability, has emerged as the predominant strategy, effectively controlling tumor progression while preserving organ function, with a low disease - specific mortality rate (11.2%, 15/134) (32).

Notably, the combination of brentuximab vedotin with CHP (cyclophosphamide, doxorubicin, and prednisone), substituting vincristine, has been demonstrated to significantly enhance both progression - free and overall survival in CD30 - expressing peripheral T - cell lymphomas when compared to conventional CHOP (33). Furthermore, allogeneic hematopoietic stem cell transplantation (allo-HSCT) has been investigated as a potentially curative approach for eligible patients, particularly those attaining remission subsequent to induction therapy. however, its application is limited by substantial costs and a high risk of transplant-related mortality (34).

**Figure 5:**
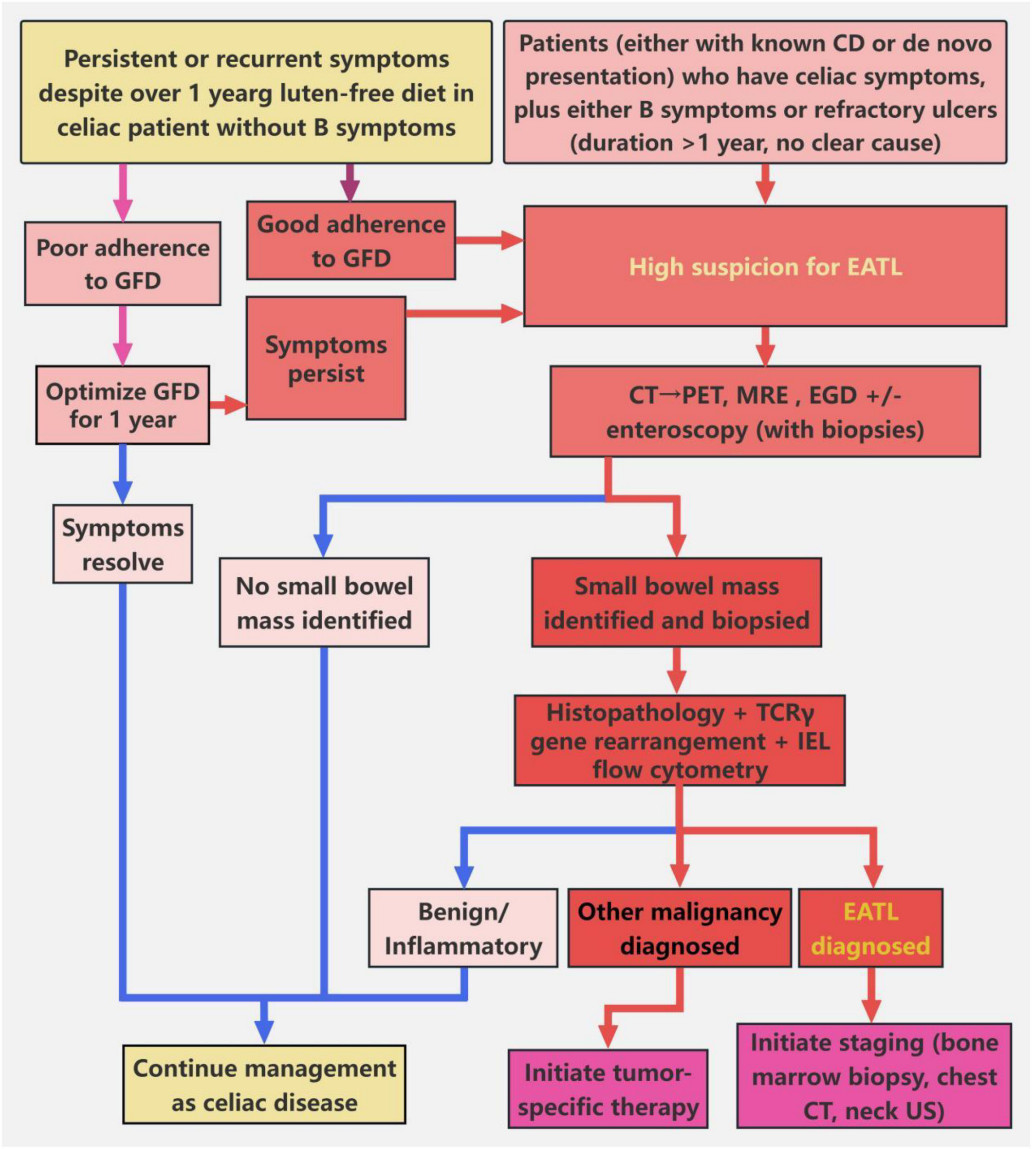
Screening algorithm for patients with suspected EATL. This algorithm guides the evaluation of suspected EATL in patients with CD or celiac-like manifestations. For CD patients with persistent or recurrent celiac symptoms despite ≥12 months of gluten-free diet (GFD), and without B symptoms (unexplained fever, night sweats, or ≥10% body weight loss), GFD adherence should first be ascertained. In cases of poor adherence, GFD optimization for 12 months is indicated; symptom resolution prompts continuation of CD management. For patients with adequate GFD adherence and persistent symptoms, suspicion for EATL is raised. In parallel, patients (either with known CD or *de novo* presentation) who have celiac symptoms, plus either B symptoms (unexplained fever, night sweats, or ≥10% body weight loss) or refractory ulcers (duration >1 year, no clear cause), also trigger high suspicion for EATL. For all patients with high EATL suspicion, noninvasive abdominal-pelvic CT is performed initially. If no suspicious small-bowel mass is identified on CT, PET-CT is subsequently obtained to detect subclinical lesions. Once a small-bowel mass is identified (either on CT or PET-CT), enteroscopy (e.g., double-balloon enteroscopy) is performed for targeted small-bowel biopsy. Collected biopsy specimens undergo histopathological assessment, combined with T-cell receptor γ (TCRγ) gene rearrangement testing (to identify clonality) and intraepithelial lymphocyte (IEL) flow cytometry (to detect aberrant phenotypes). If EATL is confirmed, staging workup (including bone marrow biopsy, chest CT, and neck ultrasonography) is initiated. If alternative malignancy is diagnosed, management proceeds per the identified condition; benign or inflammatory findings prompt resumption of CD-focused care. For patients with no detected mass (on CT/PET-CT) and non-diagnostic biopsies (when obtained), CD management is continued.

Despite aggressive interventions, the prognosis of EATL remains extremely poor. Gale et al. reported 1-year and 5-year overall survival rates of 38.7% and 9.7%, respectively, with corresponding disease-free survival rates of 19.4% and 3.2% (12). Therefore, early screening is paramount, particularly in patients presenting atypically. The current literature advocates a systematic diagnostic workup for unexplained cutaneous ulcers. This evaluation should encompass whole-body imaging (e.g., PET-CT), duodenal biopsy, serological testing for CD (anti-tTG IgA, EMA), and HLA typing (DQ2/DQ8)—even in patients lacking gastrointestinal symptoms (8, 25, 26, 35). Early deep-tissue biopsy, when coupled with comprehensive immunophenotyping, molecular analyses (e.g., T-cell receptor [TCR] gene rearrangement studies), and emerging techniques (e.g., Raman spectroscopy), may enable the early identification of occult lymphomatous malignancies (21, 23, 36) (**Figure 5**).

## Conclusion

We report a fatal case of EATL in a young male who initially presented with refractory, symmetric cutaneous ulcers and chronic diarrhea, ultimately resulting in a prolonged diagnostic delay. This case underscores the importance of considering a neoplastic etiology in the differential diagnosis of progressive, treatment-refractory dermatoses—even in younger patients. Clinicians should recognize that paraneoplastic cutaneous ulcers may represent early manifestations of occult hematologic malignancies. In patients with concomitant chronic diarrhea, a thorough evaluation to exclude underlying CD—an established precursor to EATL—is imperative. A low threshold for performing deep tissue biopsy, serological screening for celiac disease, and comprehensive systemic workup is critical for timely diagnosis. For this highly aggressive lymphoma, early recognition and prompt intervention are paramount to improving clinical outcomes.

## Data Availability

The original contributions presented in the study are included in the article/supplementary material. Further inquiries can be directed to the corresponding author.

## References

[1] Swerdlow SH Campo E Pileri SA Harris NL Stein H Siebert R . The 2016 revision of the world health organization classification of lymphoid neoplasms. Blood. (2016) 127:2375–90. doi: 10.1182/blood-2016-01-643569, PMID: 26980727 PMC4874220

[2] Malamut G Chandesris O Verkarre V Meresse B Callens C Macintyre E . Enteropathy associated t cell lymphoma in celiac disease: A large retrospective study. Dig Liver Dis. (2013) 45:377–84. doi: 10.1016/j.dld.2012.12.001, PMID: 23313469 PMC7185558

[3] Lebwohl B Green PHR Emilsson L Mårild K Söderling J Roelstraete B . Cancer risk in 47,241 individuals with celiac disease: A nationwide cohort study. Clin Gastroenterol Hepatol. (2022) 20:e111–e31. doi: 10.1016/j.cgh.2021.05.034, PMID: 34033925

[4] Delabie J Holte H Vose JM Ullrich F Jaffe ES Savage KJ . Enteropathy-associated t-cell lymphoma: Clinical and histological findings from the international peripheral t-cell lymphoma project. Blood. (2011) 118:148–55. doi: 10.1182/blood-2011-02-335216, PMID: 21566094

[5] Meeuwes FO Brink M Plattel WJ Vermaat JSP Kersten MJ Wondergem M . Enteropathy-associated t-cell lymphoma: A population-based cohort study on incidence, treatment, and outcome in the Netherlands. EJHaem. (2024) 5:1215–22. doi: 10.1002/jha2.1049, PMID: 39691269 PMC11647706

[6] Merlo G Cozzani E Canale F Miglino M Gambella M Burlando M . Cutaneous manifestations of hematologic Malignancies the experience of an italian dermatology department. Hematol Oncol. (2019) 37:285–90. doi: 10.1002/hon.2569, PMID: 30485475

[7] Younger DS Carlson A . Dermatologic aspects of systemic vasculitis. Neurol Clin. (2019) 37:465–73. doi: 10.1016/j.ncl.2019.01.017, PMID: 30952419

[8] Di Sabatino A Biagi F Gobbi PG Corazza GR . How i treat enteropathy-associated t-cell lymphoma. Blood. (2012) 119:2458–68. doi: 10.1182/blood-2011-10-385559, PMID: 22271451

[9] Khoury JD Solary E Abla O Akkari Y Alaggio R Apperley JF . The 5th edition of the world health organization classification of haematolymphoid tumours: Myeloid and histiocytic/dendritic neoplasms. leukemia. (2022) 36:1703–19. doi: 10.1038/s41375-022-01613-1, PMID: 35732831 PMC9252913

[10] Chen M Liu X Zhang Y Shi Y . Endoscopic features and clinical outcomes of enteropathy-associated t-cell lymphoma: A tertiary center retrospective study. Saudi J Gastroenterology. (2022) 28:127–34. doi: 10.4103/sjg.sjg_100_21, PMID: 34259192 PMC9007077

[11] Nijeboer P de Baaij LR Visser O Witte BI Cillessen SA Mulder CJ . Treatment response in enteropathy associated t-cell lymphoma; survival in a large multicenter cohort. Am J hematology. (2015) 90:493–8. doi: 10.1002/ajh.23992, PMID: 25716069

[12] Gale J Simmonds PD Mead GM Sweetenham JW Wright DH . Enteropathy-type intestinal t-cell lymphoma: Clinical features and treatment of 31 patients in a single center. J Clin Oncol. (2000) 18:795. doi: 10.1200/JCO.2000.18.4.795, PMID: 10673521

[13] Zhou S Chen W Lin M Chen G Chen C Huo C . Correlation of 18f-fdg pet/ct suvmax with clinical features, d-dimer and ldh in patients with primary intestinal lymphoma. J Int Med Res. (2021) 49:03000605211029809. doi: 10.1177/03000605211029809, PMID: 34250823 PMC8278467

[14] Bisig B Cairoli A Gaide O Somja J Bregnard C Gaulard P . Cutaneous presentation of enteropathy-associated t-cell lymphoma masquerading as a dusp22-rearranged cd30+ lymphoproliferation. Virchows Arch. (2022) 481:653–7. doi: 10.1007/s00428-022-03309-4, PMID: 35366115

[15] Webster A Crea P Bamford MW Hew R Griffin Y Miall F . Enteropathy-associated t-cell lymphoma presenting as cutaneous deposits. Br J Haematol. (2017) 176:7. doi: 10.1111/bjh.14375, PMID: 27766624

[16] Wu L Zhang J . Malignant lymphoma presenting as acral circulatory dysfunction: A case report. Chin J Gen Practice. (2016) 14:1969–70. doi: 10.16766/j.cnki.issn.1674-4152.2016.11.057

[17] Liang G Dai Z Liang Y Li X . Report on a rare case of type i enteropathy-associated t-cell lymphoma in a young woman and literature review. Chin J Pract Internal Med. (2023) 43:870–3. doi: 10.19538/j.nk2023100116

[18] Alvarez-Payares JC Molina A Gallo S Ramirez J Hernandez J Lopez F . Immune-mediated cutaneous paraneoplastic syndromes associated with hematologic Malignancies: Skin as a mirror of hematologic neoplasms. Cureus. (2021) 13:e19538. doi: 10.7759/cureus.19538, PMID: 34934556 PMC8668147

[19] Zhang X . A rare case of acrokeratosis paraneoplastica (bazex syndrome) and a literature review. J Int Med Res. (2025) 53:3000605251317998. doi: 10.1177/03000605251317998, PMID: 40012420 PMC11866366

[20] Ly DP Shekelle PG Song Z . Evidence for anchoring bias during physician decision-making. JAMA Intern Med. (2023) 183:818–23. doi: 10.1001/jamainternmed.2023.2366, PMID: 37358843 PMC10294014

[21] Yao X Wen G Zhou C Zhang J eds. Undiagnosed pleomorphic undifferentiated sarcoma due to superficial biopsy of a giant mass: A case report, in: 2017 National Conference on Integrated Traditional Chinese and Western Medicine in Dermatology and Venereology. (2017) Zhuhai, Guangdong, China: Chinese Association of Integrative Medicine.

[22] Důra M Štork J Felšöová A Sticová E . Diagnostic pitfalls in dermatopathology. Cesk Patol. (2023) 59:96–103., PMID: 37805266

[23] Llamas-Velasco M Paredes BE . Basic concepts in skin biopsy. Part i. Actas Dermosifiliogr. (2012) 103:12–20. doi: 10.1016/j.adengl.2011.05.005, PMID: 22459516

[24] Böer-Auer A Rose C . selection of the biopsy procedure-critical for dermatopathological diagnostics. Dermatologie (Heidelb). (2025) 76:102–14. doi: 10.1007/s00105-024-05461-1, PMID: 39836226

[25] Rubio-Tapia A Hill ID Semrad C Kelly CP Greer KB Limketkai BN . American college of gastroenterology guidelines update: Diagnosis and management of celiac disease. Am J Gastroenterol. (2023) 118:59–76. doi: 10.14309/ajg.0000000000002075, PMID: 36602836

[26] Al Somali Z Hamadani M Kharfan-Dabaja M Sureda A El Fakih R Aljurf M . Enteropathy-associated t cell lymphoma. Curr Hematol Malig Rep. (2021) 16:140–7. doi: 10.1007/s11899-021-00634-4, PMID: 34009525

[27] Iida T Nozawa H Sonoda H Toyama K Kawai K Hata K . Upfront surgery for small intestinal non-hodgkin’s lymphoma. Anticancer Res. (2020) 40:2373–7. doi: 10.21873/anticanres.14206, PMID: 32234940

[28] Shu Y Xu X Yang W Xu L . Surgery plus chemotherapy versus chemotherapy alone in primary intestinal lymphoma: A meta-analysis. J Int Med Res. (2021) 49:03000605211056845. doi: 10.1177/03000605211056845, PMID: 34763562 PMC8593296

[29] Novakonic BJ Novakonic S Frkovic-Grazio S . A single-center report on clinical features and treatment response in patients with intestinal t cell non-hodgkin’s lymphomas. Oncol Rep. (2006) 16:191–5. doi: 10.3892/or.16.1.191, PMID: 16786145

[30] Salles G Barrett M Foà R Maurer J O’Brien S Valente N . Rituximab in b-cell hematologic Malignancies: A review of 20 years of clinical experience. Adv Ther. (2017) 34:2232–73. doi: 10.1007/s12325-017-0612-x, PMID: 28983798 PMC5656728

[31] Straining R Foss F Schiffer M Amin K Agarwal S Isufi I . Real world data on efficacy and safety of epoch in t-cell lymphoma. Clin Lymphoma Myeloma Leukemia. (2025) 25:e96–e102. doi: 10.1016/j.clml.2024.09.005, PMID: 39368885

[32] Reinartz G Molavi Tabrizi C Liersch R Ullerich H Hering D Willborn K . Renaissance of radiotherapy in intestinal lymphoma? 10-year efficacy and tolerance in multimodal treatment of 134 patients: Follow-up of two german multicenter consecutive prospective phase ii trials. Oncologist. (2020) 25:e816–e32. doi: 10.1634/theoncologist.2019-0783, PMID: 32219909 PMC7216456

[33] Itodo K Md YL Facp CHPM . Expanding horizons in t-cell lymphoma therapy: Focus on personalized treatment strategies. Oncol (Williston Park NY). (2025) 39:80–4. doi: 10.46883/2025.25921036, PMID: 40117117

[34] Du J Yu D Han X Zhu L Huang Z . Comparison of allogeneic stem cell transplant and autologous stem cell transplant in refractory or relapsed peripheral t-cell lymphoma: A systematic review and meta-analysis. JAMA Netw Open. (2021) 4:e219807. doi: 10.1001/jamanetworkopen.2021.9807, PMID: 34042995 PMC8160596

[35] Al-Toma A Goerres MS Meijer JW Pena AS Crusius JB Mulder CJ . Human leukocyte antigen-dq2 homozygosity and the development of refractory celiac disease and enteropathy-associated t-cell lymphoma. Clin Gastroenterol Hepatol. (2006) 4:315–9. doi: 10.1016/j.cgh.2005.12.011, PMID: 16527694

[36] Hubbard TJE Shore A Stone N . Raman spectroscopy for rapid intra-operative margin analysis of surgically excised tumour specimens. Analyst. (2019) 144:6479–96. doi: 10.1039/c9an01163c, PMID: 31616885

